# Potential Factors Affecting Survival Differ by Run-Timing and Location: Linear Mixed-Effects Models of Pacific Salmonids (*Oncorhynchus* spp.) in the Klamath River, California

**DOI:** 10.1371/journal.pone.0098392

**Published:** 2014-05-27

**Authors:** Rebecca M. Quiñones, Marcel Holyoak, Michael L. Johnson, Peter B. Moyle

**Affiliations:** 1 Center for Watershed Sciences, University of California Davis, Davis, California, United States of America; 2 Department of Environmental Science and Policy, University of California Davis, Davis, California, United States of America; The Australian National University, Australia

## Abstract

Understanding factors influencing survival of Pacific salmonids (*Oncorhynchus* spp.) is essential to species conservation, because drivers of mortality can vary over multiple spatial and temporal scales. Although recent studies have evaluated the effects of climate, habitat quality, or resource management (e.g., hatchery operations) on salmonid recruitment and survival, a failure to look at multiple factors simultaneously leaves open questions about the relative importance of different factors. We analyzed the relationship between ten factors and survival (1980–2007) of four populations of salmonids with distinct life histories from two adjacent watersheds (Salmon and Scott rivers) in the Klamath River basin, California. The factors were ocean abundance, ocean harvest, hatchery releases, hatchery returns, Pacific Decadal Oscillation, North Pacific Gyre Oscillation, El Niño Southern Oscillation, snow depth, flow, and watershed disturbance. Permutation tests and linear mixed-effects models tested effects of factors on survival of each taxon. Potential factors affecting survival differed among taxa and between locations. Fall Chinook salmon *O. tshawytscha* survival trends appeared to be driven partially or entirely by hatchery practices. Trends in three taxa (Salmon River spring Chinook salmon, Scott River fall Chinook salmon*;* Salmon River summer steelhead trout *O. mykiss*) were also likely driven by factors subject to climatic forcing (ocean abundance, summer flow). Our findings underscore the importance of multiple factors in simultaneously driving population trends in widespread species such as anadromous salmonids. They also show that the suite of factors may differ among different taxa in the same location as well as among populations of the same taxa in different watersheds. In the Klamath basin, hatchery practices need to be reevaluated to protect wild salmonids.

## Introduction

The existence of independent local populations within a region strengthens species resiliency, the ability of a species to replenish itself after high mortality events. This is possible because local populations contribute genetic and phenotypic diversity, including diversity in life history that reflects local adaptation. In Pacific salmonids (*Oncorhynchus* spp.), such diversity in life-history traits helps to maintain populations [1] through spreading risk of extinction due to catastrophic events [2] or long periods of unfavorable conditions [3] among life-history variants. Studies suggest that populations with diverse life histories have greater stability in numbers [2] and probabilities of persistence [4], even when environmental conditions vary [5], [6]. The decline of Pacific salmonids throughout much of their range has been in part a consequence of extirpation of local populations [7] with the cumulative result of decreasing each species' adaptive capability [8]–[10]. Consequently, the ability of Pacific salmon to persist in changed environments in the future (e.g., because of climate change) requires protection of locally-adapted populations and their habitats [10]–[12].

Both environmental and anthropogenic factors (extrinsic drivers) and internal population dynamics (intrinsic drivers) determine extinction probabilities of Pacific salmon populations [13]. Extrinsic drivers of variability in Pacific salmon abundances include climatic forcing [14]–[16], freshwater habitat quality [17]–[21], and harvest by fisheries [22]–[24]. Here, we refer to climatic forcing as the effect of atmospheric phenomena on aquatic habitat conditions (e.g., winds driving upwelling events that influence prey availability in the ocean, river flow patterns). Intrinsic drivers of variability include density dependent effects from interactions with hatchery-produced conspecifics [25]–[28], cohabiting juveniles [29]–[31], and spawning adults [32]. Interactions among individuals in a population can affect trends in survival, especially when resources are limited. For salmonids, these interactions result in density dependent effects that can reduce abundance of all [33] or some [34], [35] developmental stages when habitat is limited. Deleterious density dependent effects on populations can occur in short time frames through competition and predation [31], [35], [36] or can have delayed effects on later cohorts through reduced reproduction [37], [38].

Due to paucity of data, most published studies on salmonid populations have analyzed only a small number of factors (e.g., land-use, climate change and fishing; [17]). Few, if any, studies have distinguished drivers acting on different taxon within single watersheds or among populations in the same region. For conservation, it is particularly important to know whether co-existing taxa with different life-histories are influenced by the same extrinsic and intrinsic factors, and hence whether they vary in their population dynamics and conservation needs. Here, we test the effects of ten factors measured at different spatial scales with and without time lags, as *a priori* candidate models, on three populations of Chinook salmon (*O. tshawytscha*) and one population of steelhead trout (*O. mykiss*) in two adjacent rivers. We identify and compare factors (explanatory variables) relating to climatic forcing, habitat quality, and resource management that may alter trends in survival of spring Chinook salmon, fall Chinook salmon, and summer steelhead from the Salmon River and fall Chinook salmon from the Scott River. While management practices aimed at increasing abundances of these fishes focus mainly on habitat improvement and or hatchery supplementation, in fact the factors that drive survival are not fully understood. Our study was designed to tease out which factor, or set of factors, explained the greatest amount of observed variability in survival of species for which the data existed. Given the limited availability of standardized long-term data sets, our study is designed to show which of the factors are most likely to merit further investigation and if there are indications of factors that humans can manipulate through management for long-term persistence of salmonids. Analysis of Klamath salmonid survival indices can elucidate the complexity of factors that act on widespread species with diverse life histories.

### Study Site and Taxa Investigated

**Figure 1:**
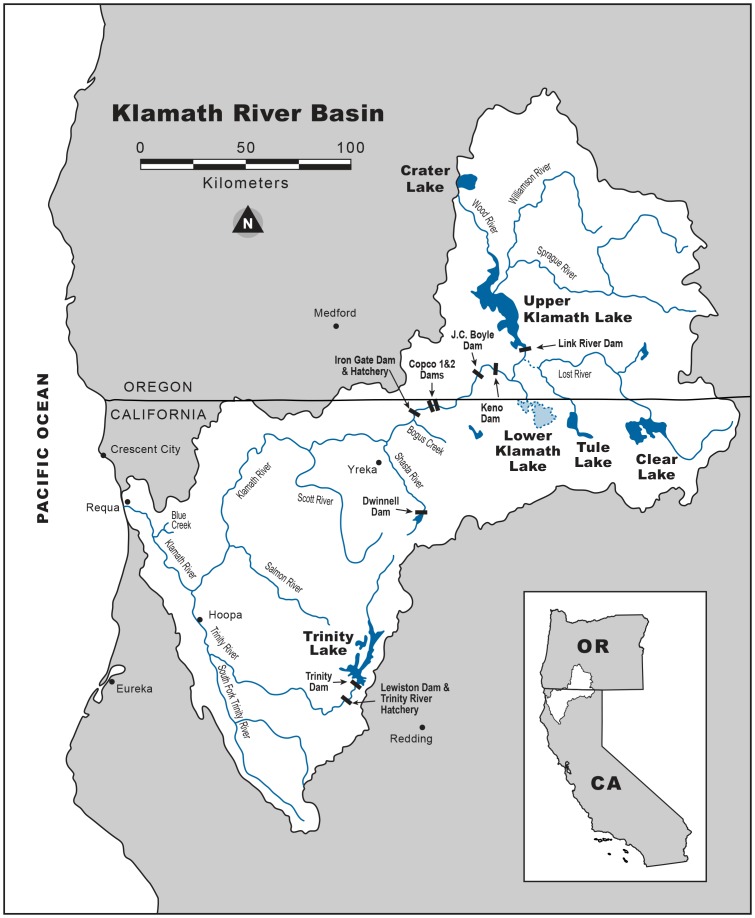
Klamath River basin, California and Oregon (modified from Quiñones et al. 2013). Rectangles represent dams.

The Klamath River of southern Oregon and northern California is the second largest river system in California, draining approximately 30,000 km^2^ (Fig. 1). Like many widespread species, Klamath River salmonids (Salmonidae) face many natural and human-caused stressors, including dams, habitat degradation, climate change and interactions with hatchery salmonids [39], [40]. The Klamath River basin once supported 55 separate taxa of salmonids, but ocean-maturing chum salmon (*O. keta*) and pink salmon (*O. gorbuscha*) are facing local extinction [22], [41] as are significant portions (∼40%) of the stream-maturing taxa, including spring Chinook salmon and summer steelhead [42]. Coho salmon (*O. kisutch*; Southern Oregon and Northern California Coasts Evolutionarily Significant Unit) were listed as threatened under the federal (62 FR 24588) and state Endangered Species Acts and are largely reliant on hatchery supplementation [43]. Two hatcheries, Iron Gate Hatchery (IGH) and Trinity River Hatchery, release Chinook salmon, coho salmon, and steelhead in the basin. Iron Gate Hatchery is located on the mainstem Klamath River approximately 300 km from the Pacific Ocean (Fig. 1). Klamath River stocks may represent the best opportunity for recovery of salmonids in California because of current efforts to restore extensive areas of habitat and plans to remove four large dams.

Previous studies have evaluated trends of spawning adult abundances in the Klamath basin (e.g., [44], [45]). Quiñones et al. [45] proposed that hatchery practices may be affecting spawning adult abundances but did not evaluate the full suite of factors that may be affecting trends. Quiñones [44], in contrast, did consider multiple factors potentially acting on abundances; abundance trends of evaluated taxa were at least partially driven by resource management, especially harvest and hatchery practices, but factors differed by taxon and location. Trends in two of the taxa appeared to also be driven by factors associated with climate change (ocean conditions, snow depth). Factors lagged up to five years appeared to significantly affect adult abundances. Time lags are important because, although most Pacific salmon-related management (e.g., harvest quotas) is completed from one year to the next, effects from drivers are often expressed at longer time scales [46], [47]. However, neither of these studies considered the confounding effect that parental abundance can have on recruitment [48] and so may not provide a clear interpretation of environmental effects on species survival.

#### Scott and Salmon River watersheds

Located in adjacent watersheds, the Salmon and Scott Rivers each drain about 2000 km^2^ and flow northwest into the Klamath River (Fig. 1). The watersheds differ greatly in land-use and aquatic habitat quality. The Salmon River watershed is among the least altered in the Klamath River basin [49], although summer temperatures in most reaches can approach salmonid tolerance levels [50]. The Salmon River is distinctive in that it continues to support natural runs of spring Chinook salmon and summer steelhead that are largely extirpated from other parts of the Klamath basin [49] and large portions of the state [40]. Streams in the Salmon River watershed are characterized by steep, bedrock-dominated channels. Flows are sustained by snowmelt from the Marble Mountains and Trinity Alps. The Salmon River watershed is ∼98% forested and managed by the Klamath and Six Rivers National Forests (Klamath National Forest, unpublished data). Land-uses in the watershed include timber harvest, grazing, and mining but to a lesser extent than in the Scott River watershed.

In contrast, the Scott River watershed has been significantly altered by channel straightening, drainage of wetlands, and hydraulic mining [51]. High summer temperatures and fine (<4 mm) sediment loads have degraded salmonid habitats [51]. Fall Chinook salmon currently make up the only significant run of anadromous salmonid, although spring Chinook and coho salmon were once numerous [39]. However, coho salmon are still present in some tributaries [40]. The mainstem river flows through a wide valley (Scott Valley) in the upper reaches and then through a bedrock-controlled canyon. Flows in the Scott River are sustained by snowmelt and groundwater inputs from the Scott Valley aquifer. Removal of water for irrigation exacerbates low base flows [52] to the extent that long stream reaches dried in about four of the last 12 years (e.g., 2009). Approximately 63% of the watershed is privately owned. Primary land-uses in the watershed are agriculture, grazing, timber harvest, and mining.

#### Chinook salmon

Chinook salmon life histories differ in age of seaward migration, length of fresh water, estuarine, and oceanic residence, marine distribution, ocean migratory patterns, and age and season of spawning migration [39], [53]. In the Klamath River, spawning migration begins as early as March (spring-run) and continues through September (fall-run) [54]. Spring Chinook salmon are reproductively immature when they enter fresh water and migrate further upstream than fall Chinook salmon [39]. Spawning by spring and fall Chinook salmon starts as early as late August and continues until December. Spring Chinook in the Salmon River normally spawn in late September or early October (B. Olson, U.S. Fish and Wildlife Service, personal communication). Mature adults are usually 2–5 years old and die shortly after spawning. The only two self-sustaining populations of spring Chinook salmon remaining in the Klamath River basin are in the Salmon and South Fork Trinity rivers. Juvenile Chinook salmon predominantly (> 80%) rear in fresh water for only a few months (∼5) before entering the ocean in the fall or early winter [54], [55]. Abundances of Salmon River and Scott River fall Chinook salmon appear to be stable, while abundances of Salmon River spring Chinook salmon appear to be increasing [45]. However, hatchery-produced conspecifics may be replacing naturally-produced spawners of at least one taxon (e.g., fall Chinook salmon), raising concerns about the viability and persistence of wild stocks [45].

#### Steelhead trout

Steelhead are the anadromous form of rainbow trout. Two distinct life-history patterns are recognized in the Klamath River basin: winter and summer steelhead [56]. Winter steelhead move upstream as mature fish after onset of winter rains between November and April, shortly before spawning. Summer steelhead move upstream as immature fish from April to June, spend the summer months in deep pools where they mature, and spawn from December to April. Their life histories differ in smolt age, length of marine residency, and patterns of reproduction [56]. Steelhead adults are capable of spawning once a year but often spawn every other year, up to four times. Adults become reproductively mature from age 1 to 5 [56]. Juveniles will spend one to two years in cold fast-flowing perennial streams and are often associated with riffle habitats. Trends of adult Iron Gate Hatchery steelhead and Salmon River summer steelhead abundances show steep declines [45] and these populations appear to be on the brink of extirpation [40], [57].

## Materials and Methods

Our goal was to use statistical models to evaluate all factors, identified from the literature, likely affecting survival of four salmonid populations. Factors included variables potentially affecting population density and habitat quality, including variables associated with climatic and hydrologic conditions (Table 1). Although factors likely interact with one another, we initially preserved their individual identities to facilitate hypothesis generation. Variables were ln transformed when necessary to meet the assumption of normality as required by initial testing (standard least squares) in order to identify candidate variables prior to model building.

**Table 1:** Name, explanation, source and transformation of variables used in modeling of salmonid survival from the Klamath River, California, USA, 1980–2012.

Category/Variable	Explanation	Source	Transformation
**Population Variables**			
Ocean abundance	Estimated number (1981–2012) of age 3 and 4 Klamath River fall Chinook	California Department of Fish and Wildlife (CDFW), pre-season report, 2012	None
Ocean harvest	Commercial harvest rate (1981–2012) on age 3 and 4 Klamath River fall Chinook	CDFW, pre-season report, 2012	Ln(OH)
IGH releases	Number of juvenile Chinook or steelhead released (1971–2009) by Iron Gate Hatchery	Iron Gate Hatchery (IGH), unpublished data, 2010	None
IGH returns	Number of adult Chinook or steelhead returning to IGH (1968–2009)	IGH, unpublished data, 2010	Ln(ln IGHr)
**Climate Variables**			
North Pacific Gyre Oscillation index (NPGO)	Average of index values for May and June	DiLorenzo 2013	None
Pacific Decadal Oscillation index (PDO)	Average of index values for May and June	Mantua 2013	None
Multivariate El Niño Oscillation iindex (MEI)	Average of index values for May and June	Wolter 2010	None
**Habitat Variables**			
Stream flow	Annual (1968–2009) base flows (July- September)	California Data Exchange Center 2010	Ln(flow)
Snow depth	April 1 snow depth on Scott Mountain (1986–2009)	Department of Water Resources, 2010	None
Equivalent Roaded Acreage index (ERA)	Watershed disturbance index (1980–2009)	Klamath National Forest, 2010	None
**Taxa**			
Salmon River spring Chinook	Number of adults observed during surveys(1980–2012)	Klamath National Forest, 2012	Ln(no. fish/km)
Salmon River fall Chinook	Number of adults (1980–2012) from carcass surveys	CDFW 2012	Ln(no. fish)
Salmon River summer steelhead	Number of adults (1980–2012) from snorkel surveys	Klamath National Forest, 2012	Ln(no. fish/km)
Scott River fall Chinook	Number of adults (1980–2012) from carcass surveys	CDFW 2012	Ln(no. fish)

### Response Variables (Taxa)

Four time series of annual spawning-adult counts (S_t_; 1981–2012) of the different taxa were obtained from the California Department of Fish and Wildlife (D. Chesney, personal communication) and Klamath National Forest (M. Meneks, unpublished data). Spring Chinook salmon and summer steelhead data were standardized as the number of fish per kilometer from annual snorkel surveys in July or August. Only adult counts were used for summer steelhead due to common misidentification of large resident trout as small steelhead (R. Quiñones, personal observation). Fall Chinook salmon data were the estimated total number of spawning adults in each river calculated from mark-recapture carcass surveys (California Department of Fish and Wildlife, unpublished data). Survey methods used to collect these data, as well as trends within the abundance time series, can be found in Table S1 in File S1.

An index of survival (ln R_t_/S_t_) for each taxon was constructed following a log-transformed Ricker stock-recruitment model commonly used for salmonid taxa [58], [59]. Survival was calculated by dividing cohort recruitment (R_t_) by the total number of spawning adults for brood year t (S_t_) (Tables S2-5 in File S1). This ratio is also referred to as apparent growth rate [60].

R_t_ was calculated as: 
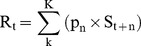
(1)where k to K is the range of ages considered as reproductively mature adults. This range was age 3 to 5 years for Chinook salmon, and age 4 to 7 years for steelhead. In the equation, p_n_ is proportion of age 3 to 7 year old fishes returning to spawn at year t and S_t+n_ is the total number of spawning adults in subsequent brood years. Age composition results based on scale (fall Chinook salmon) or otolith (steelhead) analyses were used to determine the proportion of age t+n fish needed to calculate cohort survival, as reported in agency reports [61]–[73]. Age composition for Salmon River and Scott River fall Chinook was available for 14 of 27 years (1981–2012). The proportion of age t+n Salmon River spring Chinook salmon was constructed using the same age composition as Salmon River fall Chinook because age-specific data was not found for this taxon. The age composition of steelhead collected from the Trinity, Scott, and Salmon Rivers from 2007 to 2009 (B. Hodge, personal communication) was used to determine proportions of age t+n adults of Salmon River summer steelhead. The average values of age proportions were used for years without age-specific data. Time series were ln transformed and tested for autocorrelation to meet assumptions of normality in preliminary testing.

### Population Variables

Population variables included factors that directly increase or decrease salmonid abundance and therefore may affect density dependent processes. Population variables were ocean abundance, ocean harvest rate, Iron Gate Hatchery (IGH) juvenile releases, and IGH adult returns. Analysis of population variables can also characterize effects from resource management since harvest quotas, which influence harvest rate, and hatchery operations are set by management decisions.

Commercial harvest of Klamath runs occurs immediately before they enter the river to spawn (ocean harvest). Ocean harvest rates (actual rate of catch) differ from harvest quotas (predicted catch) in that they are actual measurements of the number of fish caught over time rather than modeled projections. We used ocean harvest rate instead of total ocean catch to facilitate direct comparison because total catches reflect changes to harvest practices over time. However, we also considered the potential effect of ocean abundance to evaluate population level effects prior to harvest. Ocean harvest rate (1981–2008) is a measure of annual commercial harvest effort aimed at Klamath River fall Chinook salmon (ages 3 and 4; California Department of Fish and Game (CDFG), unpublished data). Consequently, we lagged our variable of ocean harvest rate by 3 and 4 years. The fishery, however, catches salmon from other runs and localities. Spring Chinook salmon, for example, are not distinguished from the more abundant fall Chinook salmon in the fishery [40]. IGH releases (steelhead and fall Chinook juveniles) and returns (steelhead and fall Chinook adults) specific to each species were used in the analyses. Hatchery returns were the total number of fish of each taxon that returned to the hatchery each year. We assumed that competition between wild and hatchery-reared conspecifics began upon the release of juveniles from the hatchery, since comprehensive, standardized data on the timing of hatchery releases and outmigration of wild fishes was not available at the time of our analysis. Hatchery releases were lagged by one year (t+1) to test for the effect of competition between hatchery releases and juvenile salmonids rearing in the wild.

### Climate Variables

The influence of climatic conditions on Pacific salmonid survival is well-established (e.g., [15], [74]). Climate variables are factors that influence ocean conditions (e.g., temperature, salinity), ocean productivity (e.g., plankton concentrations and abundance of other prey species) and regional weather patterns that govern precipitation and air temperatures. We chose variables that operate at large-basin (North Pacific Gyre Oscillation, NPGO; Pacific Decadal Oscillation, PDO) and regional spatial scales (El Niño Southern Oscillation, ENSO), and occur at about interannual (ENSO), decadal (NPGO), and interdecadal (PDO) time scales [15], [74], [75]. In the ocean, climatic conditions (e.g., NPGO) can alter the timing and duration of upwelling events that provide nutrients necessary for primary productivity [76]. The magnitude of upwelling events and subsequent primary production can directly affect the amount of prey (i.e. zooplankton) available to juvenile salmonids [77]. Ocean conditions, particularly food availability, at time of ocean entry by salmonid juveniles (critical period hypothesis) can heavily influence subsequent adult abundance [30]. Because we wanted to test the influence of ocean conditions at this critical period, we averaged the May and June values of the climate variables and lagged the variables by one year. Studies (e.g., [55]) have found that most salmon in these systems enter the ocean in their first spring.

After analyzing watershed-specific hydrographs, we concluded that May and June were the months when juvenile Klamath River salmonids most likely entered the ocean due to timing of peak outflows. These months are consistent with field captures of outmigrants in the basin (e.g., [78]). The Multivariate El Niño Southern Oscillation Index (MEI; 1968–2009) incorporates data of sea-level pressure, surface wind, sea surface temperature, surface air temperature, and cloud cover associated with El Niño and La Niña events [79]. These latter two events usually occur primarily in the tropics but have secondary effects in the Northern Pacific Ocean [80]. El Niño conditions are represented by significantly positive MEI values, while negative values represent La Niña conditions [79], [81]. The Pacific Decadal Oscillation (PDO) index measures monthly sea surface temperature variability in the Northern Pacific Ocean [15], [82]. PDO events usually last 20–30 years. Cool PDO regimes dominated from 1890 to 1924 and from 1947 to 1976. Warm PDO regimes dominated from 1925 to 1946 and from 1977 through about 1997 [83]. The North Pacific Gyre Oscillation index (NPGO; 1968–2009) is a measure of sea surface height variability in the Northeastern Pacific Ocean and reflects changes in salinity and nutrient concentrations in the California Current [75], [84].

### Habitat Variables

Habitat variables were factors that influence fresh water habitat quality. Habitat variables were river flow (m^3^s^−1^), snow depth (m), and Equivalent Roaded Acreage (ERA). The amount of base flow in the Salmon and Scott rivers indicates the amount of habitat available (e.g., by influencing wetted perimeter and depth) and is an indirect indicator of habitat quality (e.g., water temperature) during spawning migrations [85]. Therefore, we added the total amount of flow from July through September when evaluating flow with spring Chinook salmon and summer steelhead but flows from September through October when evaluating flows with fall Chinook salmon. Flow data was downloaded from the California Data Exchange Center (at http://cdec.water.ca.gov). Snow depth (m) on April 1 in the Klamath River basin forecasts stream flow and local climatic conditions (Natural Resources Conservation Service at http://www.wcc.nrcs.usda.gov/.html). We used April 1 snow depth (1986–2009) on Scott Mountain (A. Reising, Department of Water Resources, personal communication), which is located in the Scott River watershed, adjacent to the Salmon River watershed. We assumed that snowmelt there reflects the water yield potential and spring air temperatures in these watersheds. Snow depth at t+1 was used as proxy for rearing habitat conditions because consistent, standardized temperature data were not found for the Klamath basin. Snow depth and spring air temperatures affect the quality of stream habitat available for rearing juvenile salmonids and adult summer steelhead and spring Chinook holding during summer months. Lower snow depth and associated high air temperatures result in low flow, warm water conditions in summer because both of these systems are largely (Scott River) or wholly (Salmon River) dependent on snowmelt to feed flows until the onset of sustained rains in fall. Consequently, it was reasonable to consider the indirect effects that snow depth may have on adults due to increased mortality.

Equivalent Roaded Acreage (ERA) is used as an index of watershed disturbance from wildfire and land-use practices such as timber harvest, road building and restoration [86]. The index provides an indicator of watershed conditions by comparing the watershed-specific level of disturbance with the risk of increased peak flows that result in stream channel alteration (e.g., scouring, sedimentation; D. Elder, Klamath National Forest, personal communication). To determine ERA, impacts from wildfire and land use were standardized to road acreages that would generate the same stream alterations through the calculation of disturbance coefficients [87]. The channel sensitivity, soil erodibility, hydrologic response, and slope stability of each watershed is incorporated into each ERA value. We used ERA indices specific to the Salmon and Scott rivers for years 1980 to 2009 in our models. Indices were provided by the Klamath National Forest (G. Bousfield, Klamath National Forest, personal communication).

Although up to eleven variables were initially considered in our analysis a much smaller number (3 or less) were considered as candidates for modeling (described below), in order to preserve parsimony in model building.

### Modeling Framework

To compare variables driving survival of local populations, we first used inferential statistics (correlations) to determine individual variables that were significantly (*P*<0.05) correlated to the index of survival (Table S6 in File S1). This was done as an initial screening exercise to avoid problems associated with short time series and over-fitting due to multiple comparisons. Specifically, permutation tests were used to determine the relationship between response and explanatory variables. Permutation tests are robust to serial autocorrelation, which is typical of time-series data [88]. The resulting *P* value is the probability that reshuffling of the data will result in a test statistic (bivariate correlation) that is as or more extreme than the observed test statistic if the null hypothesis were true [89]. We shuffled our data 99,999 times, approximately two orders of magnitude more than Manly's (1997; in [89]) recommendation of 1000 permutations at a level of significance of 0.05. Permutations were completed using software available online [90]. This step reduced our ability to consider the complementarity of explanatory variables, and reflected a compromise based on the length of time series (sample size) available for analysis. The process of parsimony requires the balance of evaluating enough variables to identify the preferred model while not including so many variables that models are over-fitted. Because of this, we established criteria to limit the number of variables included (3) in each model in an unbiased manner.

### Linear Mixed-Effects Models

The five variables most significantly correlated to each taxon's survival constituted the universe of potential variables considered in building linear mixed-effects models fitted by restricted maximum likelihood. Autoregression analysis of survival indices found autocorrelations at lags of 3 years for Chinook salmon and lag of 1 year for summer steelhead. Consequently, we included “year” as a random-effect variable in our models. All other explanatory variables were considered as fixed effects. Modeling was completed using the nlme function in R statistical software (version 3.0.2; [91]).

We built models using the following conditions (as in [92]):

1. Only variables significantly (*P* <0.05) correlated with survival were used to develop candidate models.
2. Explanatory variables with correlations to one another higher than 0.5 were not used in the same model (Table S7 in File S1). Only the variable with the highest *R^2^* value was retained.
3. No more than 3 (N/10) variables were used in each model (again to reduce over-fitting of models).
4. Datasets varied between models depending on the time lags used and because we used the maximum available series length for each variable. When the data were the same, models were compared using Akaike's Information Criterion (AIC_c_). Models were treated as equivalent if ΔAIC_c_ was less than 2 [93].
5. When multiple models were equally preferred (lowest AIC_c_ or ΔAIC_c_ <2) the simplest model was chosen.

Correlations between explanatory variables and survival (Table S6 in File S1), as well as correlations among explanatory variables used in models (Table S7 in File S1), are reported in the Supporting Information.

### Density Dependence

Density dependence is a major driving force in recruitment of salmonid populations [31]. It is also possible that density-dependent processes interact with extrinsic variables to influence salmonid numbers. Density-dependence effects were analyzed from the regression of the index of survival (ln R_t_/S_t_) with the number of spawning adults (S_t_) [59], [94]. The slope of this line is considered to be a density dependent parameter (β) that measures density dependent mortality between consecutive cohorts [95]. A negative value of β can be interpreted as negative feedback density dependence and signals a decreasing population growth rate concurrent with increasing abundance.

### Ethics Statement

There are no ethical conflicts with animal use because we only used data previously collected by surveys.

### Data availability

All data sets used in this study are available at watershed.ucdavis.edu.

## Results

### Correlations and linear mixed-effects models

Only two variables were significantly correlated with Salmon River spring Chinook salmon survival (Table S6 in File S1): ocean abundance t (*P* = 0.0023, *R* ^2^ = 0.38, slope = −190215, n = 22) and summer flow t (*P* = 0.026, *R* ^2^ = 0.19, slope = 3931.06, n = 27). Comparison of a model with ocean abundance as the only variable (AIC_c_ = 94.65, n = 22) to a model of ocean abundance t and summer flow t (AIC_c_ = 116.06, n = 22) suggested the single variable model as best of the two (Table 2). A best model could not be determined between models made up solely of summer flow t or ocean abundance because the models differed in the length of their time series. Consequently, AICc values could not be directly compared between models.

**Table 2:** Preferred and alternate linear mixed-effects models of factors influencing survival of Salmon River spring Chinook salmon, Salmon River fall Chinook salmon, Salmon River summer steelhead trout, and Scott River fall Chinook salmon, 1980–2007.

Taxa/Variable	AIC_c_	*P*	N	Intercept	Slope
**Salmon River spring Chinook salmon**					
Ocean abundance t	94.65	0.0023	22	0.86	2.0 E-06
Flow t	116.14	0.026	27	−0.81	4.7 E-05
**Salmon River fall Chinook salmon**					
IGH returns t	80.54	0.016	27	15.97	−7.11
**Salmon River summer steelhead**					
Flow t	109.76	0.026	27	−1.19	4.2 E-05
**Scott River fall Chinook salmon**					
Ocean abundance t X IGH returns	100.61	0.011	22	0.74	−2.2 E-06
IGH returns t	94.26	0.046	27	16.83	−7.57

Only one variable (Table 2) was significantly correlated with Salmon River fall Chinook salmon survival: IGH returns t (*P* = 0.016, *R* ^2^ = 0.21, slope = −0.03; Table S6 in File S1).

Only one variable (Table 2) was significantly correlated with Salmon River summer steelhead survival: summer flow t (*P* = 0.024, *R* ^2^ = 0.18, slope = 4394.34; Table S6 in File S1).

Two variables were significantly correlated with Scott River fall Chinook salmon survival: IGH returns t (*P* = 0.049, *R* ^2^ = 0.15, slope = −0.02, n = 27) and ocean abundance t(*P* = 0.014, *R* ^2^ = 0.28, slope = −128481.6, n = 22; Table S6 in File S1). A comparison of single (AIC_c_ = 107.21, n  = 22) and two variable models (AIC_c_ = 100.61, n = 22) built with ocean abundance determined the two variable model as the best of the two (Table 2). A preferred model could not be determined among models made up solely of IGH returns t (AIC_c_ = 94.26, n = 27) and the two variable model due to differences in the length of time series (Table 2).

### Density dependence

**Figure 2:**
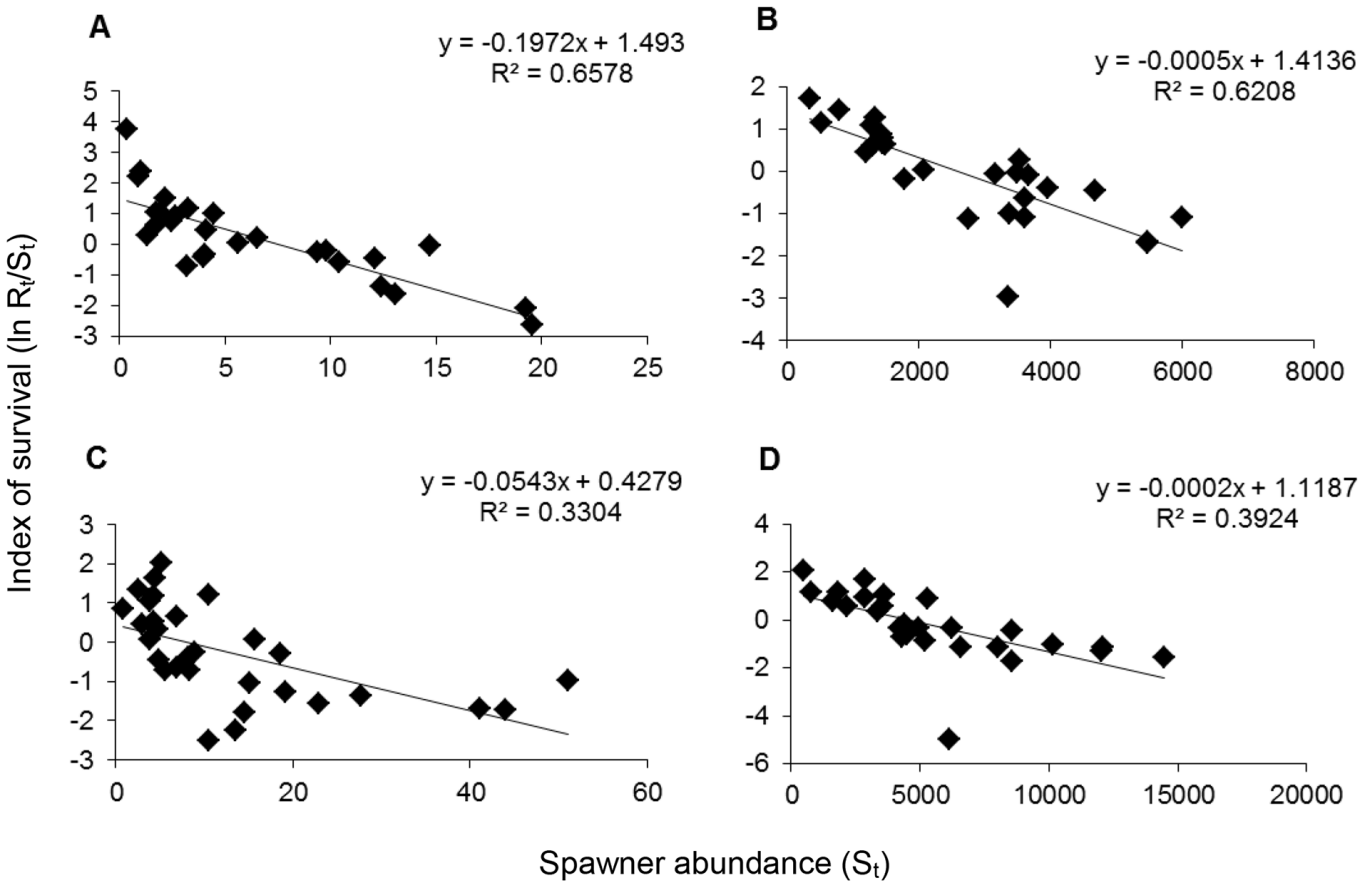
Log-transformed Ricker stock-recruitment models for four anadromous salmonids from the Klamath River basin, California. A =  Salmon River spring Chinook salmon, B = Salmon River fall Chinook salmon, C =  Salmon River summer steelhead, D =  Scott River fall Chinook salmon.

All four taxa showed significant (*P*<0.05) density-dependent effects between consecutive years (t vs. t+1), in other words, cross-annual-cohort density dependence within a generation (Fig. 2). Density dependent parameters for Salmon River spring Chinook salmon (β = −0.197, *R* ^2^ = 0.66, *P*<0.0001; Fig. 2A), Salmon River fall Chinook salmon (β = −0.0005, *R^2^* = 0.62, *P*<0.0001; Fig. 2B), Salmon River summer steelhead (β = −0.0543, *R^2^* = 0.33, *P* = 0.0009; Fig. 2C), and Scott River fall Chinook (β = −0.0002, *R^2^* = 0.39, *P* = 0.0005; Fig. 2D) were all significantly negative.

## Discussion

Both extrinsic and intrinsic factors appear to drive survival of Klamath River basin salmonids, and yet the type of variables and the timing of their effects differ among taxa. Although only three variables (IGH returns t, ocean abundance t, flow t), were statistically related to survival of the four taxa in our study, the combination of variable(s) acting on each taxon was unique. This suggests that drivers of temporal dynamics differ by both life-history and location (Table 2). The variation among taxa in these analyses emphasizes the complexity of factors driving Pacific salmon abundances.

### Hatchery returns

Hatchery returns (IGH returns t) appeared to be inversely related to survival of both Salmon River and Scott River fall Chinook salmon (Table 2, Table S6 in File S1). Interbreeding between hatchery and wild conspecifics can reduce fitness [96] and resiliency [10], [25], [97] of the population as a whole so that reduction in survival over the long-term could be expected if populations become maladapted to natural conditions. Previous work [45] suggested that hatchery operations negatively impact abundance trends of adult salmon to the point where some wild populations could be replaced by hatchery-produced conspecifics. Here, we suggest that the potential mechanism for replacement of wild populations in some watersheds is through decreased survival rates of wild populations.

Recent review of operations at IGH provided several recommendations to improve the fall Chinook salmon program [98]. One recommendation that may influence survival of fall Chinook salmon was to decrease the size of the program in order to reduce spread of disease from the myxozoan parasite, *Ceratomyxa shasta*. *C. shasta* occurs naturally in the Klamath River basin but incidence of infection is thought sufficiently high (66–88%, [99]) to significantly increase the mortality of outmigrating juvenile fall Chinook salmon [100]. Levels of the disease vector depend on the release of myxospores by decaying adult Chinook salmon carcasses [101]. Consequently, a significant decrease in number of hatchery-produced fall Chinook salmon adults could reduce rate of disease transmission in the basin.

In the Klamath basin, interactions between wild and hatchery-produced adults need to be investigated to evaluate the potential impact of hatchery fish on wild conspecifics. One clear need is to determine the proportion of in-river spawners made up by adults of hatchery origin. Although adult carcasses are checked for hatchery marks in both watersheds, not all hatchery fall Chinook are marked [98] and marks that do exist may go undetected during field surveys (R. Quiñones, personal observations). In one California fall Chinook salmon population (Mokelumne River), the proportion of in-river spawners of hatchery origin was as high as ∼90%, making wild populations a “sink” to hatchery “sources”, and likely reducing persistence of fish of wild parentage [60]. The shift in source-sink dynamics can result from a disconnect between management of population abundance and a clear understanding of population productivity [60], including measures of survival and recruitment.

### Summer flows

Our results support the notion that abundances of taxa with extended stream rearing (spring Chinook salmon, summer steelhead) are significantly influenced by freshwater habitat conditions (as in [102]). Both spring Chinook salmon and summer steelhead survival appeared to be positively correlated to Salmon River summer (July, August, September) flows (flows t; Table S6 in File S1). Flow was the single variable retained in alternate models for both taxa (Table 2). Survival of the two taxa, thus, appears to increase or decrease with summer flow.

Spring Chinook salmon and summer steelhead spend more time in fresh water than other Klamath River anadromous salmonids [54], [56], including fall Chinook salmon, so watershed conditions, especially high water temperatures associated with low flows, are likely to have a strong influence on survival. Both taxa are reproductively immature when they enter rivers [39] in summer and need cold pools to reside in during the summer months [40]. Limited number of deep pools due to low summer flow can result in decreases in egg survival due to increased exposure of maturing fish to warm temperatures [103]. This is especially a problem in the Salmon River, which has a limited amount of cold pool habitat [49] so spring Chinook salmon and summer steelhead may directly compete for its use. One focus of future work should be to determine if there are sufficient deep pools in the Salmon River to support both spring Chinook salmon and summer steelhead in years with different summer flows.

The Salmon River hydrograph depicts a flow regime typical of snowmelt-fed systems in Mediterranean climates, with flows peaking in late fall or early winter and mostly decreasing until there is a smaller peak in spring due to snowmelt; this is followed by decreasing flows through summer and fall. Consequently, flows are low during spawning migrations of spring Chinook salmon and summer steelhead. Coupled with high air temperatures and paucity of riparian vegetation to provide shade, low flow conditions in summer can be the most stressful for fishes in the Salmon River. To this end the Salmon River has been formally recognized (Federal Clean Water Act section 303d listing) as having water quality impaired due to high temperatures (http://www.waterboards.ca.gov).

Because the Salmon River is a snowmelt-fed system that is unregulated, it would seem that little could be done to mitigate the effect of low flows on spring Chinook salmon and summer steelhead. Several stakeholders have recommended timber harvest as one way to augment flows in the watershed (R. Quiñones, personal observation). One study did show timber harvest (both clear-cut and partial harvest) reduced evapotranspiration sufficiently to increase water yields [104]. However, the impacts were temporary, felt only during the snow deposition and snowmelt periods (November through June), and had no significant effect during summer months. Risks associated with timber harvest (e.g., increased erosion) may outweigh any temporary benefits to fish from increased Salmon River flows.

Because high water temperatures resulting from low flows are already a factor reducing survival of spring Chinook salmon and summer steelhead, these taxa may be particularly vulnerable to conditions expected with climate change, including a decrease in summer flows and increases in air temperatures [44]. Whether ongoing measures, such as streamside tree planting to provide shade and protection of alpine meadows to increase summer flows, are sufficient to offset expected climate change impacts will need to be assessed.

### Ocean abundance

Ocean abundance was inversely related to survival of Salmon River spring Chinook salmon and Scott River fall Chinook salmon in our analyses (Table S6 in File S1). However, models for each taxon differed in that ocean abundance t was the sole variable retained in an alternate model for spring Chinook salmon but was retained together with IGH returns in the alternate model for Scott River fall Chinook (Table 2).

Inter- and intraspecific ocean abundance of Pacific salmon has been shown to result in density-dependent effects that can affect survival, particularly during early and late marine developmental stages (reviewed in [105]). The effect on survival is particularly acute when ocean prey is limited for both the juvenile salmon and their predators [25], [106]. When prey is scarce, juvenile salmon may experience relatively high size-mediated predation and decreased survival (reviewed in [105]). Conversely, when prey are readily available, survival and abundance of juvenile salmon at sea can increase through rapid growth and reduced predation. This increase in juvenile abundance can later result in density-dependent growth of maturing adults and subsequently lower reproductive success because egg size and fecundity are directly correlated with fish size (reviewed in [105]). Future work could determine the relative abundance of salmon from different populations, perhaps through isotopic analysis.

There is growing evidence that the ocean's carrying capacity may be exceeded during years of low productivity due to increasing inputs of juvenile salmon from hatcheries [25], [106]–[109]. An alternate model for Scott River fall Chinook salmon survival comprised of ocean abundance t and IGH returns t suggests that this mechanism affects this population. The marine distribution of Scott River fall Chinook salmon and cohabitating hatchery salmon as well as relative availability of resources needs to be determined to evaluate this relationship. Alternatively, analysis of size class frequencies could help elucidate if this is the case for Scott River fall Chinook. Reduction in size at age has been associated with reduction of the ocean's carrying capacity and with density-dependent effects on recruitment and survival [108], [110]–[111].

### Density dependence

While releases of large numbers of hatchery fish is one mechanism by which density dependence may affect Klamath River salmonids while at sea, density dependent population growth can potentially affect wild salmonids in all habitats by mediating growth, movement, reproduction, and survival [112]. Our calculations of density dependence parameters from Ricker stock-recruitment models were indicative of compensatory density dependence (-β) for all four taxa, as expected for most fishes [112]. Although errors associated with field measurements can limit utility of these measurements [112], [113], here we briefly discuss how compensatory density dependence can affect survival of four Klamath River salmonid populations.

Compensatory density dependent processes result in increased mortality (decreased survival) when abundances are high and vice versa. Compensatory density dependence characterizes mortality from spawning to fry emergence in salmonids (e.g., [114]) and mortality of immature life history stages in general [115]. As discussed above, low flows and associated high temperatures may be impairing egg development and reproductive success of spring Chinook salmon and summer steelhead in the Salmon River. Successful spawning, incubation, and emergence in the Scott River, in contrast, may be curtailed by the lack of suitable spawning substrates. Pacific salmon need loosely packed, gravel in which to build their nests (redds; [116]). Even small amounts of fine (<4 mm) sediment deposition in the interstitial spaces of gravel substrates can impair successful incubation and emergence of larval salmonids from redds [117]. Fine sediment loads in both watersheds can exceed acceptable levels (Klamath National Forest, unpublished data) and spawning areas in the Scott River have been declared impaired due to heavy accumulation of fine sediments in the stream channel [51]. Although sediment levels are not as high in the Salmon River, loads of fine sediment still embed otherwise suitable gravel beds in some areas (Klamath National Forest, unpublished data).

Compensatory density-dependent processes may help rebuild or maintain fall Chinook salmon populations since decreasing trends were not detected by time series analysis of abundances [45]. However, recent estimates of adult spring Chinook salmon and summer steelhead depict significantly lower numbers compared to historical levels. Spring Chinook salmon are believed to have once been numerically dominant in the Klamath River basin, potentially numbering 100,000 individual adults in any given year [39], [40]. Today, a couple of thousand adults, at most, return each year to the South Fork Trinity and Salmon rivers (Klamath National Forest, unpublished data; [118]), about a 98% long-term decline in numbers. Summer steelhead are thought to have been abundant in cold-water tributaries throughout the Klamath basin but now are represented by a small fraction of historical numbers [40]. Compensatory density-dependent processes, therefore, may not help rebuild critically low abundances of spring Chinook salmon and summer steelhead populations if other mechanisms such as Allee effects are inhibiting population growth. Contrary to expectations from compensation, populations of these taxa do not appear to be growing at small abundances; Achord et al. [119] found density-dependence to have adverse effects on a population, through increases in mortality, even at low abundance levels. Population growth of small or declining populations may become negative (a.k.a. critical depensation) when average recruitment rates cannot compensate for mortality rates [118]. Population characteristics (e.g., Allee effects; [120]), fish behavior, harvest practices, and changes to food webs can all lead to critical depensation [121]–[123].

Although both of these taxa persist, incessantly low recruitment and survival likely threatens their recovery. Holt [124] found that the probability of recovery by an imperiled species is more threatened by recurring low survival (as defined by R_t_/S_t_) than from increasingly frequent die-offs. Ultimately, the concept of density dependence is closely tied to that of carrying capacity [125]. Knowing the carrying capacity of the habitats used by Klamath River salmonids will help inform decisions regarding harvest quotas, escapement targets, and hatchery goals that could dictate survival of local populations.

## Conclusions

Our results show that different runs (populations) of salmon and steelhead are affected by different combinations of factors in different locations; these factors include likely interactions with other runs and species. This complexity is not surprising because it reflects both local adaptation and the ability of co-occurring anadromous salmonids to maximize resource utilization. Our study comes with the caveat that we studied only four populations of many in the Klamath Basin and did not have ways to measure effects of a number of other variables, such as predation or interspecific competition. Recognition of this high complexity makes management of diverse salmonids using the same and nearby watersheds more difficult because results of management actions may be less predictable than assumed. The implication of our results to salmonid conservation is that there is no one measure that may protect all local populations of salmonids even if they inhabit the same watershed or are part of the same metapopulation. Recovery efforts and resource management may need to be tailored to address the stressors specifically faced by separate populations in order to effectively protect larger taxanomic groups such as Evolutionarily Significant Units.

Our analyses also suggest research that is needed to improve management in the Klamath River basin, especially to resolve some ambiguities in our findings. For example, questions needing answers for our particular system include: (a) Is spawning gravel a limiting resource in some watersheds? (b) What is the proportion of fish straying from hatcheries in naturally spawning populations? And, (c) does competition between summer steelhead and spring Chinook salmon limit abundance of either species?

## Supporting Information

File S1 **Supporting tables.** Table S1, Trends of four anadromous salmonids taxa from the Klamath River basin, California, based on linear regressions coupled with randomized permutations (n =  99,999; modified from Quiñones et al. 2013). Table S2, Salmon River spring Chinook salmon stock-recruitment data set. Table S3, Salmon River fall Chinook salmon stock-recruitment data set. Table S4, Salmon River summer steelhead stock-recruitment data set. Table S5, Scott River fall Chinook salmon stock-recruitment data set. Table S6, Slope, *R* ^2^, and *P* values of correlations between salmonid survival and variables. Superscripts specify species or location of variable. c =  Chinook salmon, s =  steelhead, sal  =  Salmon River, sc  =  Scott River. For example, ^c,s^ IGH releases t specifies that Chinook salmon or steelhead data were used depending on the time series being analyzed. Significant values (*P*<0.05) are in bold. Table S7, Coefficient of determination (*R* ^2^) for correlations of variables significant to Salmon River spring Chinook salmon and Scott River Chinook salmon survival (ln).
(DOCX)
