# Supplementary material for: Potential Factors Affecting Survival Differ by Run-Timing and Location: Linear Mixed-Effects Models of Pacific Salmonids (Oncorhynchus spp.) in the Klamath River, California
Source: PLoS One. 2014 May 27;9(5):e98392. doi: 10.1371/journal.pone.0098392 (PMC4035341; doi:10.1371/journal.pone.0098392)
Supplement: File S1 — Supporting tables. Table S1, Trends of four anadromous salmonids taxa from the Klamath River basin, California, based on linear regressions coupled with randomized permutations (n = 99,999; modified from Quiñones et al. 2013). Table S2, Salmon River spring Chinook salmon stock-recruitment data set. Table S3, Salmon River fall Chinook salmon stock-recruitment data set. Table S4, Salmon River summer steelhead stock-recruitment data set. Table S5, Scott River fall Chinook salmon stock-recruitment data set. Table S6, Slope, R 2, and P values of correlations between salmonid survival and variables. Superscripts specify species or location of variable. c = Chinook salmon, s = steelhead, sal = Salmon River, sc = Scott River. For example, c,s IGH releases t specifies that Chinook salmon or steelhead data were used depending on the time series being analyzed. Significant values (P<0.05) are in bold. Table S7, Coefficient of determination (R 2) for correlations of variables significant to Salmon River spring Chinook salmon and Scott River Chinook salmon survival (ln). (DOCX) [file pone.0098392.s001.docx]

**Table S1.** Trends of four anadromous salmonids taxa from the Klamath River basin, California, based on linear regressions coupled with randomized permutations (n = 99,999; modified from Quiñones et al. 2013).

| **Taxon** | **Direction of trend** | **Years of collection** | **No. years analyzed** | **Survey method(s)** | **Data source** |
| --- | --- | --- | --- | --- | --- |
| Salmon River spring Chinook salmon | Increasing* | 1968-2009 | 34 | Snorkel surveys | KNF |
| Salmon River fall Chinook salmon | Decreasing | 1978-2009 | 32 | Carcass and redd surveys | CDFW |
| Salmon River summer steelhead | Decreasing* | 1968-2009 | 34 | Snorkel surveys | KNF |
| Scott River fall Chinook salmon | Decreasing | 1978-2009 | 32 | Carcass and redd surveys, video weir | CDFW |

**P* values significant at α = 0.05, KNF = Klamath National Forest, CDFW = California Department of Fish and Wildlife.

**Table S2.** Salmon River spring Chinook salmon stock-recruitment data set.

| **Brood Year** | **R_3_** | **R_4_** | **R_5_** | **R_t_** | **S_t_*** | **R/S** | **Ln R/S** |
| --- | --- | --- | --- | --- | --- | --- | --- |
| 1981 | 0.41 | 0.74 | 0.59 | 1.73 | 1.29 | 1.34 | 0.29 |
| 1982 | 0.99 | 4.69 | 0.06 | 5.74 | 5.62 | 1.02 | 0.02 |
| 1983 | 12.52 | 1.10 | 0.39 | 14.01 | 0.33 | 42.02 | 3.74 |
| 1984 | 1.46 | 6.64 | 0.04 | 8.14 | 0.89 | 9.16 | 2.21 |
| 1985 | 8.86 | 0.60 | 0.03 | 9.50 | 2.14 | 4.43 | 1.49 |
| 1986 | 0.80 | 0.59 | 0.04 | 1.43 | 19.56 | 0.07 | -2.61 |
| 1987 | 0.79 | 0.66 | 0.08 | 1.53 | 3.18 | 0.48 | -0.73 |
| 1988 | 0.88 | 1.42 | 0.07 | 2.36 | 19.27 | 0.12 | -2.10 |
| 1989 | 1.89 | 1.11 | 0.19 | 3.20 | 1.75 | 1.83 | 0.60 |
| 1990 | 1.48 | 3.22 | 0.08 | 4.78 | 1.72 | 2.79 | 1.03 |
| 1991 | 4.29 | 0.76 | 0.25 | 5.31 | 1.91 | 2.78 | 1.02 |
| 1992 | 1.97 | 4.28 | 0.27 | 6.52 | 4.12 | 1.58 | 0.46 |
| 1993 | 5.71 | 4.49 | 0.08 | 10.29 | 3.23 | 3.19 | 1.16 |
| 1994 | 6.00 | 1.06 | 0.04 | 7.10 | 9.34 | 0.76 | -0.27 |
| 1995 | 1.29 | 1.57 | 0.05 | 2.91 | 4.02 | 0.72 | -0.32 |
| 1996 | 2.24 | 0.84 | 0 | 3.08 | 12.42 | 0.25 | -1.39 |
| 1997 | 1.12 | 1.39 | 0 | 2.52 | 13.04 | 0.19 | -1.65 |
| 1998 | 1.70 | 4.91 | 0.06 | 6.67 | 2.64 | 2.53 | 0.93 |
| 1999 | 4.63 | 7.30 | 0.04 | 11.97 | 4.48 | 2.67 | 0.98 |
| 2000 | 4.46 | 0.66 | 0 | 5.12 | 2.44 | 2.10 | 0.74 |
| 2001 | 0.16 | 2.40 | 0 | 2.56 | 3.98 | 0.64 | -0.44 |
| 2002 | 5.94 | 1.82 | 0.30 | 8.06 | 9.82 | 0.82 | -0.20 |
| 2003 | 2.21 | 5.07 | 0.19 | 7.47 | 12.09 | 0.62 | -0.48 |
| 2004 | 6.77 | 3.26 | 1.08 | 11.12 | 1.03 | 10.81 | 2.38 |
| 2005 | 4.36 | 1.37 | 0 | 5.73 | 10.42 | 0.55 | -0.60 |
| 2006 | 3.40 | 4.56 | 0 | 7.96 | 6.50 | 1.23 | 0.20 |
| 2007 | 8.39 | 5.60 | 0 | 13.99 | 14.71 | 0.95 | -0.05 |
|  |  |  |  |  |  |  |  |

*Spawner abundance was standardized as number of adults per kilometer because survey lengths varied among years.

**Table S3.** Salmon River fall Chinook salmon stock-recruitment data set.

| **Brood year** | **R_3_** | **R_4_** | **R_5_** | **R_t_** | **S_t_** | **R/S** | **Ln R/S** |
| --- | --- | --- | --- | --- | --- | --- | --- |
| 1981 | 663.16 | 1090.40 | 109.95 | 1863.51 | 1200 | 1.55 | 0.44 |
| 1982 | 1455.09 | 879.60 | 80.42 | 2415.11 | 1300 | 1.86 | 0.62 |
| 1983 | 2345.60 | 1361.28 | 73.30 | 3780.17 | 1275 | 2.96 | 1.09 |
| 1984 | 1816.56 | 1240.66 | 73.50 | 3130.72 | 1442 | 2.17 | 0.78 |
| 1985 | 1655.60 | 1244.10 | 95.02 | 2994.72 | 3164 | 0.95 | -0.05 |
| 1986 | 1660.20 | 1608.37 | 30.13 | 3298.70 | 3665 | 0.90 | -0.11 |
| 1987 | 2146.30 | 510.05 | 26.98 | 2683.32 | 3950 | 0.68 | -0.39 |
| 1988 | 680.63 | 456.63 | 71.93 | 1209.20 | 3600 | 0.34 | -1.09 |
| 1989 | 609.35 | 1217.57 | 71.12 | 1898.04 | 3610 | 0.53 | -0.64 |
| 1990 | 1624.79 | 1203.78 | 109.50 | 2938.07 | 4667 | 0.63 | -0.46 |
| 1991 | 1606.39 | 1040.25 | 111.23 | 2757.87 | 1480 | 1.86 | 0.62 |
| 1992 | 2682.75 | 1882.70 | 122.16 | 4687.61 | 1325 | 3.54 | 1.26 |
| 1993 | 2512.37 | 2067.76 | 43.59 | 4623.72 | 3533 | 1.31 | 0.27 |
| 1994 | 2759.33 | 581.20 | 7.80 | 3348.33 | 3493 | 0.96 | -0.04 |
| 1995 | 711.97 | 273.00 | 36.08 | 1021.05 | 5475 | 0.19 | -1.68 |
| 1996 | 390.00 | 610.68 | 0 | 1000.68 | 5463 | 0.18 | -1.70 |
| 1997 | 814.92 | 1174.00 | 0 | 1988.92 | 6000 | 0.33 | -1.10 |
| 1998 | 1433.00 | 1374.00 | 17.00 | 2824.00 | 1453 | 1.94 | 0.66 |
| 1999 | 1296.00 | 2039.00 | 13.32 | 3348.32 | 780 | 4.29 | 1.46 |
| 2000 | 1245.00 | 213.12 | 0 | 1458.12 | 1772 | 0.82 | -0.19 |
| 2001 | 53.28 | 116.38 | 0 | 169.66 | 3350 | 0.05 | -2.98 |
| 2002 | 288.42 | 579.32 | 29.16 | 896.90 | 2747 | 0.33 | -1.12 |
| 2003 | 703.46 | 493.51 | 48.84 | 1245.81 | 3375 | 0.37 | -1.00 |
| 2004 | 658.56 | 826.76 | 408.00 | 1893.32 | 333 | 5.69 | 1.74 |
| 2005 | 1103.27 | 516.80 | 0 | 1620.07 | 506 | 3.20 | 1.16 |
| 2006 | 1278.40 | 878.54 | 0 | 2156.94 | 2069 | 1.04 | 0.04 |
| 2007 | 1615.38 | 1812.69 | 0 | 3428.07 | 1432 | 2.39 | 0.87 |

**Table S4.** Salmon River summer steelhead stock-recruitment data set.

| **Brood year** | **R_4_** | **R_5_** | **R_6_** | **R_7_** | **R_t_** | **S_t_*** | **R/S** | **Ln R/S** |
| --- | --- | --- | --- | --- | --- | --- | --- | --- |
| 1977 | 13.11 | 5.68 | 0.09 | 0.02 | 18.90 | 51 | 0.37 | -0.99 |
| 1978 | 34.43 | 0.60 | 0.10 | 0.07 | 35.20 | 10.50 | 3.35 | 1.21 |
| 1979 | 3.63 | 0.71 | 0.37 | 0.02 | 4.72 | 22.86 | 0.21 | -1.58 |
| 1980 | 4.28 | 2.57 | 0.09 | 0.08 | 7.03 | 27.60 | 0.25 | -1.37 |
| 1981 | 15.59 | 0.66 | 0.38 | 0.17 | 16.80 | 15.65 | 1.07 | 0.07 |
| 1982 | 3.98 | 2.64 | 0.87 | 0.06 | 7.55 | 41.09 | 0.18 | -1.69 |
| 1983 | 16.01 | 6.08 | 0.30 | 0.03 | 22.41 | 4.33 | 5.17 | 1.64 |
| 1984 | 36.85 | 2.09 | 0.14 | 0.02 | 39.10 | 5.11 | 7.65 | 2.03 |
| 1985 | 12.64 | 0.96 | 0.10 | 0.03 | 13.73 | 18.61 | 0.74 | -0.30 |
| 1986 | 5.80 | 0.68 | 0.17 | 0.02 | 6.67 | 4.75 | 1.40 | 0.34 |
| 1987 | 4.10 | 1.15 | 0.11 | 0.01 | 5.38 | 19.10 | 0.28 | -1.27 |
| 1988 | 6.98 | 0.78 | 0.07 | 0.01 | 7.84 | 43.98 | 0.18 | -1.72 |
| 1989 | 4.70 | 0.52 | 0.06 | 0.02 | 5.29 | 15.09 | 0.35 | -1.05 |
| 1990 | 3.16 | 0.41 | 0.09 | 0.01 | 3.66 | 6.93 | 0.53 | -0.64 |
| 1991 | 2.45 | 0.59 | 0.05 | 0.02 | 3.11 | 4.89 | 0.64 | -0.45 |
| 1992 | 3.60 | 0.34 | 0.08 | 0.02 | 4.03 | 8.33 | 0.48 | -0.73 |
| 1993 | 2.07 | 0.53 | 0.08 | 0.03 | 2.71 | 5.61 | 0.48 | -0.73 |
| 1994 | 3.22 | 0.58 | 0.14 | 0.04 | 3.97 | 3.77 | 1.05 | 0.05 |
| 1995 | 3.51 | 0.95 | 0.18 | 0.04 | 4.69 | 2.93 | 1.60 | 0.47 |
| 1996 | 5.78 | 1.24 | 0.21 | 0.05 | 7.28 | 4.29 | 1.70 | 0.53 |
| 1997 | 7.49 | 1.45 | 0.27 | 0.06 | 9.26 | 2.47 | 3.74 | 1.32 |
| 1998 | 8.79 | 1.87 | 0.29 | 0.03 | 10.98 | 3.84 | 2.86 | 1.05 |
| 1999 | 11.32 | 2.01 | 0.16 | 0 | 13.49 | 4.19 | 3.22 | 1.17 |
| 2000 | 12.16 | 1.12 | 0.01 | 0 | 13.30 | 6.90 | 1.93 | 0.66 |
| 2001 | 6.79 | 0.10 | 0.02 | 0.01 | 6.93 | 8.93 | 0.78 | -0.25 |
| 2002 | 0.62 | 0.17 | 0.04 | 0.02 | 0.85 | 10.49 | 0.08 | -2.51 |
| 2003 | 1.04 | 0.25 | 0.12 | 0.01 | 1.42 | 13.51 | 0.10 | -2.25 |
| 2004 | 1.52 | 0.85 | 0.03 | 0.01 | 2.41 | 14.51 | 0.17 | -1.79 |
| 2005 | 5.14 | 0.23 | 0.04 | 0.01 | 5.41 | 8.11 | 0.67 | -0.40 |
| 2006 | 1.39 | 0.28 | 0.03 | 0 | 1.71 | 0.74 | 2.29 | 0.83 |
|  |  |  |  |  |  |  |  |  |

*Spawner abundance was standardized as number of adults per kilometer because survey lengths varied among years.

**Table S5.** Scott River fall Chinook salmon stock-recruitment data set.

| **Brood year** | **R_3_** | **R_4_** | **R_5_** | **R_t_** | **S_t_** | **R/S** | **Ln R/S** |
| --- | --- | --- | --- | --- | --- | --- | --- |
| 1981 | 871.22 | 1264.11 | 0 | 2135.33 | 6556 | 0.33 | -1.12 |
| 1982 | 2132.33 | 1045.33 | 499.10 | 3676.76 | 10176 | 0.36 | -1.02 |
| 1983 | 3698.86 | 2456.53 | 302.98 | 6458.37 | 3568 | 1.81 | 0.59 |
| 1984 | 4143.72 | 1491.24 | 244.01 | 5878.98 | 1801 | 3.26 | 1.18 |
| 1985 | 2515.45 | 1201.02 | 94.10 | 3810.57 | 4408 | 0.86 | -0.15 |
| 1986 | 2025.90 | 463.14 | 126.14 | 2615.19 | 8041 | 0.33 | -1.12 |
| 1987 | 781.24 | 620.87 | 165.36 | 1567.47 | 8566 | 0.18 | -1.70 |
| 1988 | 1047.30 | 813.87 | 308.81 | 2169.98 | 5200 | 0.42 | -0.87 |
| 1989 | 1372.86 | 1519.92 | 166.81 | 3059.59 | 4188 | 0.73 | -0.31 |
| 1990 | 2563.82 | 821.04 | 144.77 | 3529.64 | 1615 | 2.19 | 0.78 |
| 1991 | 1384.95 | 1302.93 | 1107.60 | 3795.48 | 2165 | 1.75 | 0.56 |
| 1992 | 9265.28 | 5451.51 | 783.84 | 15500.63 | 2838 | 5.46 | 1.70 |
| 1993 | 9195.70 | 3858.01 | 133.08 | 13186.79 | 5300 | 2.49 | 0.91 |
| 1994 | 6507.76 | 998.10 | 35.84 | 7541.70 | 2863 | 2.63 | 0.97 |
| 1995 | 1929.66 | 788.48 | 364.33 | 3082.47 | 14477 | 0.21 | -1.55 |
| 1996 | 2186.24 | 1793.22 | 23.00 | 4002.46 | 12097 | 0.33 | -1.11 |
| 1997 | 3024.83 | 2293.00 | 127.00 | 5444.83 | 8561 | 0.64 | -0.45 |
| 1998 | 3083.00 | 1656.00 | 30.00 | 4769.00 | 3327 | 1.43 | 0.36 |
| 1999 | 2479.00 | 7559.00 | 247.51 | 10285.51 | 3584 | 2.87 | 1.05 |
| 2000 | 4399.00 | 154.11 | 52.92 | 4606.03 | 6253 | 0.74 | -0.31 |
| 2001 | 42.03 | 0 | 0 | 42.03 | 6142 | 0.01 | -4.98 |
| 2002 | 642.60 | 1240.00 | 262.49 | 2145.09 | 4308 | 0.50 | -0.70 |
| 2003 | 1736.00 | 1291.93 | 272.27 | 3300.20 | 12053 | 0.27 | -1.30 |
| 2004 | 2179.25 | 1340.11 | 265.32 | 3784.68 | 467 | 8.10 | 2.09 |
| 2005 | 2260.52 | 110.55 | 0 | 2371.07 | 756 | 3.14 | 1.14 |
| 2006 | 1790.91 | 1705.44 | 0 | 3496.35 | 4960 | 0.70 | -0.35 |
| 2007 | 401.28 | 2042.77 | 0 | 2444.05 | 4505 | 0.54 | -0.61 |

**Table S6.** Slope, *R*^2^, and *P* values of correlations between salmonid survival and variables. Superscripts specify species or location of variable. c = Chinook salmon, s = steelhead, sal = Salmon River, sc = Scott River. For example, c,s IGH releases t specifies that Chinook salmon or steelhead data were used depending on the time series being analyzed. Significant values (*P* < 0.05) are in bold.

|  |  | **Salmon River spring Chinook** | | | | | | |  | **Salmon River fall Chinook** | | | | |  | **Salmon River summer steelhead** | | |  | **Scott River fall Chinook** | | | |
| --- | --- | --- | --- | --- | --- | --- | --- | --- | --- | --- | --- | --- | --- | --- | --- | --- | --- | --- | --- | --- | --- | --- | --- |
| Variable | slope | | *R*^2^ | | | | *P* | slope | | | *R*^2^ | | | *P* | slope | | *R*^2^ | *P* | slope | | *R*^2^ | | *P* |
| ^c^ Ocean harvest t+3 | 0.099 | | 0.023 | | | | 0.47 | 0.238 | | | 0.11 | | | 0.11 | NA | |  |  | 0.16 | | | 0.087 | 0.16 |
| ^c^ Ocean harvest t+4 | 0.014 | | | 0.00041 | | | 0.93 | 0.199 | | | 0.078 | | | 0.20 | NA | |  |  | 0.18 | | | 0.098 | 0.15 |
| ^sal,sc^ Flow t | 3931.06 | | | | 0.19 | | **0.026** | 1690.9 | | | | 0.096 | | 0.11 | 4394.3 | | 0.18 | **0.025** | 302.88 | | | 0.028 | 0.37 |
| ^c,s^ IGH returns t | -0.015 | | | 0.082 | | | 0.15 | -0.03 | | | | 0.21 | | **0.016** | -0.066 | | 0.087 | 0.14 | -0.02 | | | 0.15 | **0.049** |
| ^c,s^ IGH releases t+1 | -594035 | | | | 0.081 | | 0.15 | -744241 | | | | | 0.08 | 0.14 | -28434 | | 0.13 | 0.073 | -262195 | | | 0.016 | 0.51 |
| ^sal, sc^ ERA t | -39.15 | | | 0.015 | | | 0.54 | -90.36 | | | | 0.05 | | 0.25 | -15.93 | | 0.002 | 0.82 | 15.44 | | | 0.013 | 0.58 |
| Snow t+1 | -1.82 | | | 0.03 | | | 0.45 | 0.26 | | | | 0.0008 | | 0.90 | 1.99 | | 0.052 | 0.34 | -1.43 | | | 0.037 | 0.4 |
| NPGO t | NA | | |  | | |  | NA | | | |  | |  | -0.1 | | 0.012 | 0.58 | NA | | |  |  |
| NPGO t+1 | 0.009 | | | 0.00013 | | | 0.96 | -0.24 | | | | 0.06 | | 0.23 | NA | |  |  | -0.23 | | | 0.086 | 0.15 |
| MEI t | NA | | |  | | |  | NA | | | |  | |  | 0.055 | | 0.007 | 0.69 | NA | | |  |  |
| MEI t+1 | 0.094 | | | 0.025 | | | 0.42 | 0.20 | | | | 0.08 | | 0.16 | NA | |  |  | 0.19 | | | 0.11 | 0.096 |
| PDO t | NA | | |  | | |  | NA | | | |  | |  | 0.19 | | 0.061 | 0.22 | NA | | |  |  |
| PDO t+1 | -0.073 | | | 0.011 | | | 0.60 | 0.036 | | | | 0.002 | | 0.83 | NA | |  |  | 0.09 | | | 0.019 | 0.5 |
| ^c^Ocean abundance t | -190215 | | | | 0.38 | **0.0023** | | -125573 | | | | | 0.16 | 0.063 | NA | |  |  | -128482 | | | 0.28 | **0.014** |

**Table S7.** Coefficient of determination (*R^2^*) for correlations of variables significant to Salmon River spring Chinook salmon and Scott River Chinook salmon survival (ln).

| **Taxon/Variables** | ***R*^2^** | **Adjusted *R^2^*** |
| --- | --- | --- |
| Salmon River spring Chinook salmon |  |  |
| Flow t x ocean abundance t | 0.016 | -0.034 |
|  |  |  |
| Scott River fall Chinook salmon |  |  |
| IGH returns t x ocean abundance t | 0.29 | 0.26 |
|  |  |  |
